# Comparative Genomics Platform and Phylogenetic Analysis of Fungal Laccases and Multi-Copper Oxidases

**DOI:** 10.1080/12298093.2020.1816151

**Published:** 2020-09-11

**Authors:** Jiayao Wu, Jaeyoung Choi, Fred O. Asiegbu, Yong-Hwan Lee

**Affiliations:** a Department of Forest Sciences, University of Helsinki, Helsinki, Finland; b Smart Farm Research Center, Korea Institute of Science and Technology, Gangneung, Republic of Korea; c Department of Agricultural Biotechnology, Center for Fungal Genetic Resources, Plant Immunity Research Center, and Research Institute of Agriculture and Life Sciences, Seoul National University, Seoul, Republic of Korea

**Keywords:** Laccase, multicopper oxidase, database, phylogenetic analysis, fungal genome

## Abstract

Laccases (EC 1.10.3.2), a group of multi-copper oxidases (MCOs), play multiple biological functions and widely exist in many species. Fungal laccases have been extensively studied for their industrial applications, however, there was no database specially focused on fungal laccases. To provide a comparative genomics platform for fungal laccases, we have developed a comparative genomics platform for laccases and MCOs (http://laccase.riceblast.snu.ac.kr/). Based on protein domain profiles of characterized sequences, 3,571 laccases were predicted from 690 genomes including 253 fungi. The number of putative laccases and their properties exhibited dynamic distribution across the taxonomy. A total of 505 laccases from 68 genomes were selected and subjected to phylogenetic analysis. As a result, four clades comprised of nine subclades were phylogenetically grouped by their putative functions and analyzed at the sequence level. Our work would provide a workbench for putative laccases mainly focused on the fungal kingdom as well as a new perspective in the identification and classification of putative laccases and MCOs.

## 1. Introduction

Laccases (EC 1.10.3.2), the biggest subgroup of multi-copper oxidases (MCOs), are known to catalyze the oxidation of a broad range of substrates such as phenolic compounds and aromatic amines [1]. Laccases have been actively investigated for their ability in the degradation of a variety of compounds, such as phenols, ascorbates, amines, lignin, and phosphates. Laccases incorporate at least two copper centers, a mononuclear T1 copper center for the oxidation of substrate and a trinuclear including one T2 and two T3 copper ions for reduction of oxygen into water [2]. Laccases could be classified by spectroscopy; “blue” laccases exhibit an absorption band at 610 nm, while “white/yellow” laccases do not show such significant absorbance [3–5].

Laccases are commonly found in organisms from bacteria to higher eukaryotes [6,7]. Typical laccases are known to be three-domain MCOs, however, two-domain laccase-like enzymes have been discovered in archaeal species, suggesting the evolutionary origin of laccases [8]. CotA, a laccase in *Bacillus subtilis*, for example, played additional roles as bilirubin oxidase activity and in brown pigmentation in spore coat [9,10]. Laccases were also known to be involved in ectopic lignin polymerization and seed coat browning in *Arabidopsis thaliana* [11,12]. In other plant species *Liriodendron tulipifera,* laccases were reported to have putative roles in iron uptake or weak electron acceptor in cytokinin degradation [13,14].

Within the fungal kingdom, laccases were known to be dynamically associated with regulatory functions at diverse developmental stages. A laccase-encoding gene *lac4* in edible straw mushroom, *Volvariella volvacea*, was induced and strongly expressed during fruit body formation [15]. Increased activity of a secretory laccase LCC1 in *Hypsizygus marmoreus* was involved in mycelial growth and primordium initiation, whereas the decreased activity of secretory laccases was observed in *Pleurotus tuber-regium* when mycelia gradually became mature [16,17]. Transcription of a laccase gene in wild-type *Neurospora crassa* was initiated during the sexual differentiation stage, while its activity in a recombined strain was only achievable when copper ions were added [18]. Fungal genes encoding laccases were also inducible to deal with different conditions. Laccase genes in *Aspergillus nidulans* were transcribed upon carbon starvation and highly induced during sexual development [19]. In addition, laccase genes in *Lentinula edodes* were expressed to resist low temperature and osmotic pressure [20].

A number of fungal laccases were known to be involved in biosynthesis of pigments. For instance, laccases were involved in green pigment synthesis in conidial spores of *Trichoderma atroviride* and *T. harzianum* [21]. A laccase encoded by *yA* in *A. nidulans* was reported to oxidize a yellow precursor into mature green pigment during asexual development, while another one encoded by *TilA* was expressed at the growing fungal hyphal tip only after spore germination and associated with melanin biosynthesis [22,23]. In *L. edodes*, laccases were also involved in melanin synthesis during developmental stages in fruiting body formation [24]. In addition, laccases were also involved in the biosynthesis of different types of melanin in *Sinorhizobium meliloti* [25].

It has been reported that laccases of white-rot fungi exhibited capability in lignin degradation aided by their high redox potential [1,26]. During the cultivation of two soft-rot ascomycetes, *Xylaria polymorpha* and *X. hypoxylon*, on the beech-wood, laccases were identified as secreted lignin-modifying enzymes [27]. Missing with lignin- or manganese-type peroxidase, a white-rot fungus *Pycnoporus cinnabarinus* possibly contains alternative pathways of lignin degradation by secreting laccase during growth in synthetic liquid media [28]. The activity of fungal extracellular laccase was also found to be changed across mycelia during their interactions between white- and brown-rot basidiomycetes from different wood decay stages as well as fungal interactions [29].

Many efforts were also made to express or engineer laccases to improve industrial performance, for example, separation and degradation of lignin in the pulp and paper industry [30–32]. There have been a number of other applications including textile, bio-energy, bioremediation, food, and pharmaceutical industries [33,34]. In the future, laccase would possibly attract more interests as “eco-friendly” enzyme for the fact that its only by-product is water [35,36].

There have been databases aimed to classify MCOs and laccase-like proteins, such as Laccase Engineering Database (LccED; http://www.lcced.uni-stuttgart.de/) based on sequence homology and LacSubPred (http://lacsubpred.bioinfo.ucr.edu/) using physicochemical properties [37,38]. To the best of our knowledge, there is no database dedicated to fungal laccases and their classification. To attain this end, we present the Fungal Laccase Database (http://laccase.riceblast.snu.ac.kr/) which would serve as a database mainly focused on fungal species based on protein domain profiles. In-depth phylogenetic analysis using the predicted laccases and MCOs would also provide a new perspective on the functional classification of fungal laccases.

## 2. Materials and methods

### 2.1. Establishment of protein domain profiles and collection of proteomes and characterized sequences

**Figure 1.**
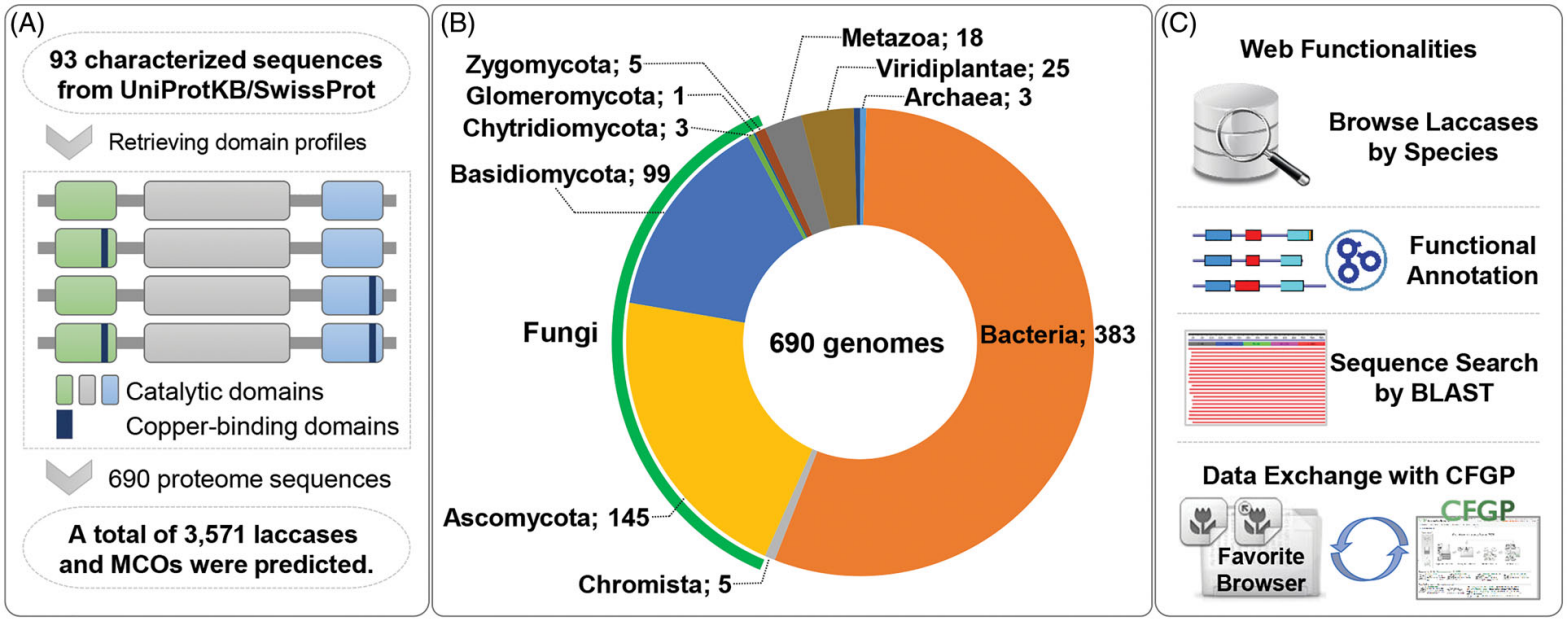
Construction of the database. (A) A schematic diagram of a three-step pipeline; (i) to collect characterized sequences from UniprotKB/SwissProt Database, (ii) to define domain profiles from the characterized sequences, and (iii) 690 proteome sequences were scanned by the pipeline; (B) Taxonomy profile of genomes included in the database. The kingdom Fungi was broken down into five phyla and indicated by the outermost green arc; (C) Web functionalities.

A total of 93 protein sequences of laccases were obtained from UniProtKB/SwissProt Database to determine domain profiles of fungal laccases by using InterProScan (version 58.0) [39,40]. The commonly shared domain profile was applied to proteome sequences for the prediction of putative laccases and MCOs, supporting the previously reported structural relationships (Figure 1 and Supplementary Table 1) [41]. In total, 3,571 laccases predicted from 690 genomes of 472 species were archived and freely available at Fungal Laccase Database (http://laccase.riceblast.snu.ac.kr/). Proteome sequences were obtained from the standardized genome data warehouse built in the Comparative Fungal Genomics Platform (CFGP 2.0; http://cfgp.snu.ac.kr/) [42].

### 2.2. Prediction of secretory laccases

To achieve better accuracy, prediction of secretory laccases was carried out by the protocol using SignalP, TMHMM, Wolf PSORT, Phobius, and PS-Scan as described previously [43]. Signal peptides were scanned by SignalP 4.1 and Phobius [44,45]. Subsequently, transmembrane helices were predicted by TMHMM, and then subcellular localization of each sequence was determined by WoLF PSORT [46,47]. Proteins containing ER-targeting signals were removed by PS-Scan (PROSITE: PS00014) [48]. Protein sequences were considered as secretory only if all tools predicted them as extracellular (Supplementary Figure 1). The default parameters were applied to all the tools as follows: Phobius, WolfPSORT, PS-Scan, SignalP 4.1, and TMHMM [44–48].

### 2.3. Motif analysis

The two protein domains of InterPro, IPR033138 (Multicopper oxidases, conserved site) and IPR002355 (Multicopper oxidase, copper-binding site), were defined based on PROSITE patterns PS00079 (Multicopper oxidases signature 1) and PS00080 (Multicopper oxidases signature 2), respectively [49]. These PROSITE signatures were scanned and analyzed to see the distribution of the patterns in predicted proteins. Conservation in protein sequences was visualized by using WebLogo [50].

### 2.4. Multiple sequence alignment and phylogenetic analysis

Multiple sequence alignments of the predicted sequences were conducted by MUSCLE, followed by the construction of neighbor-joining trees with 1,000 bootstrap replicates by MEGA [51,52]. The phylogenetic tree was visualized by using FigTree [53]. Functional annotation of the predicted laccases and MCOs was inferred from the characterized ones (Supplementary Table 2).

## 3. Results

### 3.1. Summary of Fungal Laccase Database

Based on the domain analysis by InterProScan, laccases, and MCOs were predicted by using the three key domains from 690 genomes including 253 fungal genomes (Figure 1(A)). There were 145 ascomycetes and 99 basidiomycetes accounting for the majority of the fungal genomes. Nine fungal genomes were also searched for laccases, covering underrepresented fungal phyla, that is, Chytridiomycota, Glomeromycota, and Zygomycota. In addition, genome sequences of 383 bacteria, 5 oomycetes, 3 archaea, 25 plants, and 18 metazoa were also included for comparative analysis (Figure 1(B)). As a result, a total of 3,571 genes were predicted from the genome sequences. All predicted protein sequences contained three essential domains: multicopper oxidase, type 1 (IPR001117); multicopper oxidase, type 2 (IPR011706), and multicopper oxidase, type 3 (IPR011707). In addition, there were two additional domains frequently found in the predicted laccases and MCOs that were assumed to be copper-binding motifs: multicopper oxidase, copper-binding site (IPR002355) and multicopper oxidases, conserved site (IPR033138).

In general, prokaryotes had a single putative laccase-encoding gene while eukaryotes contained multiple copies, showing a wide range of the average number per genome across the taxonomy (Table 1). Three archaeal genomes had only one putative laccase gene, while 1.5 laccases were found in bacterial genomes, on average. Oomycetes were predicted to encode four laccases on average, ranging from one to seven. In the kingdom Viridiplantae, there were significantly more laccase genes (35.1 per genomes) than any other kingdoms. Meanwhile, there were much fewer laccase genes in the kingdom Metazoa (3.3 per genome). In the kingdom Fungi, 8.4 genes encoding laccases were predicted on average. Ascomycetes and basidiomycetes contained relatively higher numbers of laccase genes, 8.2 and 9.3 per genome, respectively. Lower fungi belonging to phyla Zygomycota and Chytridiomycota encoded 4.8 and 2.7 genes per genome, respectively. The number of laccase genes in each genome seemed to be mostly dependent on taxonomy. Species belonging to the subphylum Pezizomycotina contained the highest number of laccase genes than other fungal subphyla (9.9 per genome), followed by Agaricomycotina (9.5), Pucciniomycotina (9.0), and Ustilaginomycotina (5.3). Interestingly, most species in the subphylum Saccharomycotina possessed three laccase genes per genome, while all species in Taphrinomycotina possessed only one.

**Table 1.** Statistics of laccase-encoding genes across the taxonomy.

Kingdom	Phylum	Subphylum	Count	Average number*
Archaea			3	1.0 (1)
Bacteria			383	1.5 (1–4)
Chromista			6	4.0 (1–7)
Fungi	Ascomycota	Pezizomycotina	124	9.9 (1–26)
		Saccharomycotina	33	3.2 (1–8)
		Taphrinomycotina	7	1.0 (1)
	Basidiomycota	Agaricomycotina	89	9.5 (1–20)
		Pucciniomycotina	6	9.0 (1–19)
		Ustilaginomycotina	3	5.3 (4–6)
		Wallemiomycetes	1	3.0 (3)
	Chytridiomycota	Chytridiomycetes	3	2.7 (2–3)
	Glomeromycota	Glomeromycetes	1	5.0 (5)
	Zygomycota	Mucoromycotina	5	4.8 (4–5)
Metazoa			18	3.3 (1–9)
Viridiplantae			30	35.1 (1–79)

*The lowest and highest numbers of laccase genes for each taxon were shown in the parenthesis.

### 3.2. A representative dataset

A representative dataset was defined to dissect laccases and MCOs by mainly focusing on fungal species and other microbial species for comparative analysis. The dataset was comprised of 505 laccase sequences collected from the genome sequences of 60 fungi, 3 oomycetes, and 5 bacteria (Supplementary Table 3). These genomes were selected to cover a wide range of microbial taxonomy and diverse lifestyles including plant/human pathogens, saprobes, and brown-/white-rot fungi. Nearly half of laccases turned out to be secretory proteins, 245 out of 505 (Supplementary Figure 1). Bacterial laccases seemed to be intracellular while the majority of oomycete laccases were predicted to be secretory (80.00%). Within the fungal kingdom, laccases from the phylum Zygomycota exhibited high secretory potential, four out of five, while none was secretory in Chytridiomycota. The proportion of secretory laccases was higher in Basidiomycota (60.9%) than that of Ascomycota (47.7%). When compared at the subphylum level, only 12.0% of laccases from Saccharomycotina turned out to be extracellular, while 80 out of 117 in Agaricomycotina, 7 out of 24 in Pucciniomycotina, and 5 out of 10 in Ustilaginomycotina were predicted to be secretory.

Interestingly there was a plant-specific protein domain, IPR017761 (Laccase). On the other hand, IPR017762 (Multicopper oxidase, fungi), a characteristic of fungal ascorbate oxidases, existed in 4.2% of the putative laccases from the representative dataset. In the representative dataset, 67.1% and 70.5% of the sequences had IPR002355 (Multicopper oxidase, copper-binding site) and IPR033138 (Multicopper oxidases, conserved site), respectively.

### 3.3. Phylogenetic analysis of putative fungal laccases

**Figure 2.**
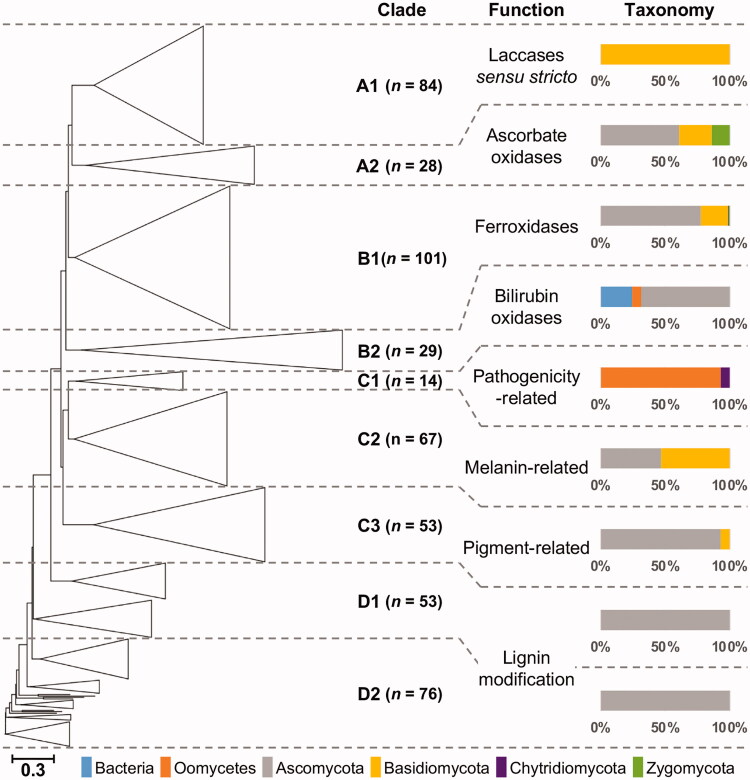
Phylogenetic illustration of the 505 predicted laccases/MCOs in the representative dataset. (Sub)clades were collapsed as triangles where applicable. Classification of (sub)clades, molecular function, and taxonomic distribution were displayed alongside of the phylogenetic tree. The number of sequences for each subclade was shown in parenthesis next to the clade notation.

Phylogenetic trees constructed by using the representative dataset were divided into four groups and named as clades A, B, C, and D. The four clades were further subdivided into nine subclades. The clade A was clustered as a sister taxon to subclade B1, with subclade B2 in a basal lineage. A larger clade comprising the clade A and B was clustered as a sister taxon to clade C, where clade D formed the most basal lineage (Figure 2).

When divided by molecular functions, subclade A1 was comprised of laccases *sensu stricto* and subclade A2 with putative fungal ascorbic oxidases (Figure 2). All the members in the subclade A1 were found in the species belonging to the phylum Agaricomycotina, including white- and brown-rot fungi. These laccases showed high sequence similarity and represented the shortest average length of protein sequences, 529 aa, among all clades. Laccases POX1 and POX2 in a lignin-degrading fungus *Pleurotus ostreatus* were found in this clade, supporting the fact that laccases *sensu stricto* were exclusively found in Agaricomycotina [5]. Lcc1 and Lcc2 in *Agaricus bisporus*, a saprophytic fungus capable of lignin degradation, were also located in the subclade A1 [54]. In addition, multiple laccases in *Fomitopsis pinicola*, a brown-rot fungus with lignin degradation ability, were grouped with the aforementioned proteins [55]. Interestingly, laccases predicted from plant pathogens were scattered across the entire tree but found only a few in the subclade A1. However, 14 out of 18 laccases of *Heterobasidion irregulare* were located in this subclade with laccases from rot fungi. It might be due to the dual lifestyle of *H. irregulare*, that is, a saprotrophic white-rot fungus and a pathogen to pine trees. Putative ascorbate oxidases (EC 1.10.3.3) were clustered to form the subclade A2 alongside with the subclade A1. Notably, ascorbate oxidases were largely secretory (17 out of 28), and sequences identified from plant pathogens outnumbered by the ones from species of other lifestyles. Several plant pathogenic species were found in the subclade A2 including *Fusarium graminearum*, *F. oxysporum*, *F. verticillioides*, and *Magnaporthe oryzae*. In addition, four out of five species belonging to Zygomycota, and several basidiomycetes were also found in the same clade. A characterized ascorbate oxidase (AO1, Pa_4_3640) from saprobic fungus *Podospora anserina* was located in this clade, suggesting a molecular function of this subclade [56].

Clade B showed broader taxonomic coverage than other clades, including bacteria, oomycetes as well as fungi (Figure 2). The majority of the characterized ferroxidases or iron transport proteins were located in subclade B1, such as ABR1 and FET3 in *P. anserina* and FET3 in *F. graminearum* [56,57] (Supplementary Table 2). Fet3p in *Saccharomyces cerevisiae* was grouped together with other ferroxidases in *Candida albicans*, *Kluyveromyces lactis,* and *Ashbya gossypii*, while laccases from fission yeast were located separately. Basidiomycete laccases also had iron uptake function [58], and formed a small subclade containing laccases found in white-/brown-rot. With two exceptions, 25 out of 27 predicted MCOs from the species belonging to the phyla Saccharomycotina and Taphrinomycotina were found in the subclade B1. It might imply that the molecular function of the MCOs in these phyla were primarily ferroxidases. It also suggests that copper-dependent iron transport by ferroxidase might be essential for all fungal species. Meanwhile, 77 out of 101 laccases in this clade were predicted to be non-secretory. Next to the ferroxidases, 7 bilirubin oxidases from bacteria, 2 oomycetes, and 20 ascomycetes formed subclade B2. Bacterial laccases, including CueO and CotA from *Escherichia coli*, together with many bilirubin oxidases from ascomycetes were grouped together in this subclade [59].

Clade C was the largest clade consisted of 134 sequences and divided into three subclades C1-3 (Figure 2). Members of the subclade C1 was identified from plant pathogenic oomycetes, with one exception, showing high secretory potential (92.3%). Laccases thought to be related to melanin synthesis were clustered into subclade C2, where 13 out of 67 laccases turned out to be secretory. Interestingly, laccases were grouped separately by their taxonomic origin, ascomycetes, and basidiomycetes. CNLAC1 and CNLAC2, virulence factors in a human pathogen *Cryptococcus neoformans*, were known to be involved in the biosynthesis of melanin [60]. They clustered with laccases of basidiomycetes to form a small group within the subclade C2. In subclade C3, the majority belonged to ascomycetes together with a few basidiomycetes (Figure 2). Some members in the subclade C3 were reported to be involved in pigment biosynthesis, implying the molecular function of the proteins in this subclade; for example, *A. nidulans* Laccase-1 or yA required for conidial green pigment and TilA in the hyphal tip and *F. graminearum* GIP1 for the biosynthesis of the mycotoxin aurofusarin [61].

The clade D, distant from the laccases *sensu stricto*, was considered as lignin-related or lignin-modifying, but not including the ones directly degrading lignin. Deletion mutants of *lac6* and *lac8* in *P. anserina* lost their ability to degrade lignin and cellulose [62]. In addition, LAC2_PODAS, a homolog of LAC1_NEURC in *N. crassa*, was also located in the same clade, implying that they might be also involved in lignin degradation [63]. Laccases LAC1-3 from *Botrytis cinerea*, a fungus able to catalyze the oxidation of lignin model compounds [64], were located in the same clade as well. Subclade D1 contained much less secretory proteins (20 out of 53), while 73.7% of proteins in subclade D2 was predicted to be secretory. TpcJ in *Aspergillus fumigatus* was the only characterized one in the subclade D1, which was specifically expressed in conidia and involved in the biosynthesis of trypacidin, a mycotoxin produced during the infection process [65].

### 3.4. Comparative analysis on copper-binding motifs

**Figure 3.**
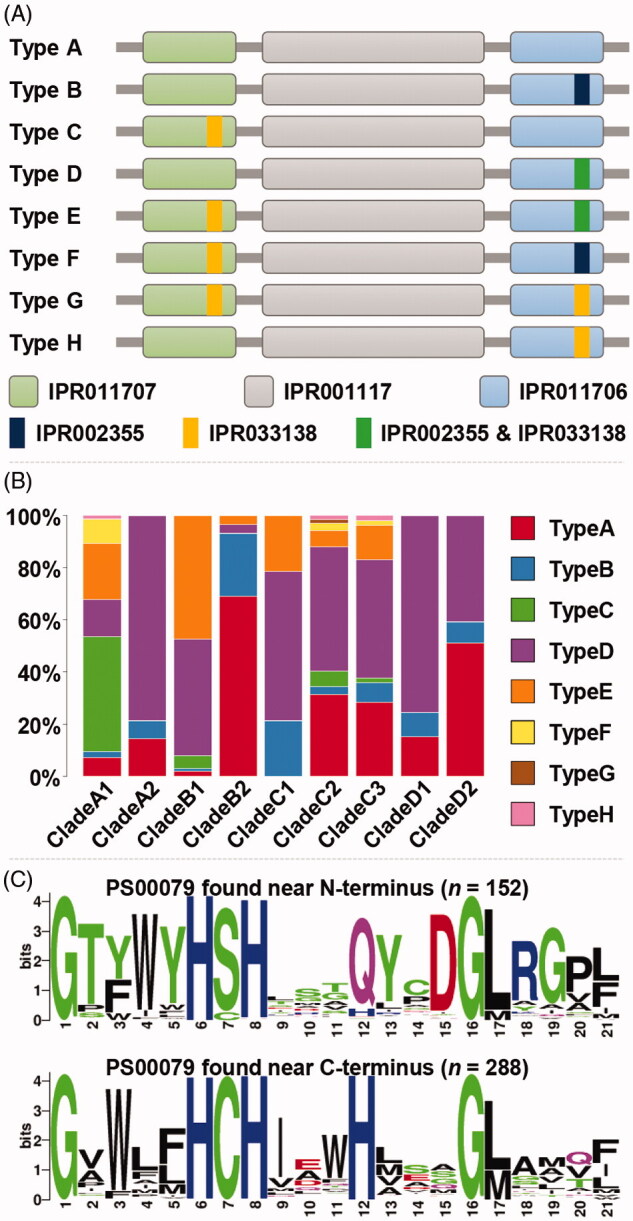
Distribution and sequence analysis of copper-binding domains in MCOs. (A) Schematic diagrams for domain profiles identified in the 505 protein sequences. Catalytic domains; IPR011707 for “Multicopper oxidase, type 3”, IPR001117 for “Multicopper oxidase, type 1”, and IPR011706 for “Multicopper oxidase, type 2”. Copper-binding domains: IPR002355 for “Multicopper oxidase, copper-binding site” and IPR033138 for “Multicopper oxidases, conserved site”; (B) Distribution of the domain profiles across the subclades; (C) Differential sequence conservation in IPR033138 between the ones near N-terminus and C-terminus.

A total of 8 domain profiles were identified from the 505 protein sequences in the representative dataset (Figure 3(A)). The majority of proteins (390 out of 505) were predicted to have at least one copper-binding motifs. Notably, the members in subclades A2, D1, and D2 did not have any copper-binding motifs near the N-terminus. Type D was the most frequently found domain profile, followed by Type A and E. Meanwhile 37 out of 47 Type C proteins were found in the subclade A1, showing subclade-specific distribution (Supplementary Table 3).

In the subclade A1, 78 out of the 84 laccases had one or two copper-binding motifs (IPR002355 and/or IPR033138). There were 75 motifs close to the N-terminus than C-terminus, while 19 were near the C-terminus. Compared to the subclade A1, fungal ascorbate oxidases in the subclade A2 had a single copper-binging site at C-terminus or no copper-binding sites (Figure 3). Another notable feature was that many ferroxidases (48 out of 101) in the subclade B1 contained two copper-binding sites at both ends of the sequences, while 46 sequences contained a binding site only at C-terminus. There were five sequences contained the binding site at N-terminal end and two sequences had neither. Interestingly, 99 out of 101 sequences in the subclade B1 contained at least one copper-binding site. In contrast, 20 out of 29 protein sequences in the subclade B2 did not have any copper-binding site. A total of 21 proteins were clustered with two characterized laccases in *C. neoformans* within the subclade C2. The majority of them (17 out of 21) did not contain copper-binding sites, while 94 out of the rest sequences in the clade C possessed one or two binding sites (Supplementary Table 3). It suggests that certain basidiomycete laccases may be involved in pigment synthesis including melanin, forming a phylogenetically separate clade. Among the clade D, most of the sequences (82 out of 129) contained single copper-binding sites at the C-terminus, while the rest did not have any (Figure 3(B)). In subclade D2, 73.7% sequences were secretory proteins and there were 39 sequences without copper-binding sites.

A total of 440 copper-binding motifs, IPR033138, were found in 358 out of 505 sequences; 152 motifs close to N-terminus and 288 near N-terminus. Since the definition of the domain was based on a PROSITE pattern, PS00079, the conservation of sequences was assessed at the amino acid level. Motifs captured by PS00079 showed a clear difference between the ones located close to N-terminus and C-terminus, showing only a handful of key residues were highly conserved for the motifs at both locations (Figure 3(C)).

### 3.5. Web utility

Fungal Laccase Database (http://laccase.riceblast.snu.ac.kr/) adopted the Data-driven User Interface powered by the CFGP 2.0 [42]. All putative laccase genes identified by the pipeline were stored by species and available in both fasta and csv format in the Browse Data page. The statistics page under “Browse by Species” gives a kingdom-/phylum-level summary, providing a glimpse into the macro-taxonomic distribution of putative genes encoding laccases and MCOs. Information on laccase scanned by the HMM profiles of the Laccase Engineering Database was also included in the database. The detail view page for each gene contains gene structure, domain profiles, GO terms, functional prediction including sub-cellular location, hydropathic plots, and sequence information. Fungal Laccase Database also provides a BLAST search function [66] in the “Tool” menu, and “Favorite Browser” which supports a personalized virtual storage and analysis hub that is synced with CFGP 2.0 where more bioinformatics tools are available (Figure 1(C)).

## 4. Discussion

Although laccases account for the largest family of multi-copper oxidase superfamily, accurate definition of laccases still remains elusive [26,67]. Previous studies discussed the minor differences in the laccase sequences between Ascomycota and Basidiomycota, and observed the low identity in sequences even within the same species [68]. This diversity and complexity have hampered the establishment of classification rules and functional categorization. Here, we have developed a new prediction pipeline and performed phylogenetic analysis for laccases and MCOs by encompassing a wide range of the kingdom Fungi.

The clades A-D were classified by more of their functions rather than their taxonomy, while the subclades were formed according to the phylum-level taxonomy. Certain subgroups of laccases were only found in a limited range of fungi, such as in the case of budding and fission yeast, with the exception of the two sequences located in the subclade C2. Ascorbate oxidases (EC 1.10.3.3) were more closely related to laccases *sensu stricto* than other MCOs [41]. POX2 from *P. ostreatus* was reported to play roles in fruiting body development with substrates other than L-ascorbic acid [5,69]. A laccase found in *F. graminearum*, aurL2, was known to be involved in the biosynthesis of aurofusarin [70], showing the functional diversity of MCOs in the subclade A2. Bilirubin oxidases, the subclade B2, were grouped together next to the ferroxidases and found in a limited range of taxa where none identified in the species belonging to the phylum Basidiomycota (Figure 2 and Supplementary Table 3). Comparing to neighboring clades, these two small groups of MCOs possessed different properties, for example, different positions of copper-binding sites. Meanwhile, a previous study reported that the phylogeny of laccases and MCOs did not exactly follow that of corresponding species [71]. It was coincided with our results, suggesting that they might have been through a wide range of functional diversification in evolutionary trajectories.

The presence and absence of copper-binding motifs showed dynamic distribution across the subclades. For example, 97.0% of members in the subclade B1 contained at least one IPR033138 domain, while only 6.9% in the subclade B2 (Supplementary Table 3). Interestingly, the target domains close to N-terminus and C-terminus showed different consensus at the protein sequence level, suggesting functional diversification of a single motif (Figure 3(C)). In addition, 284 out of 288 sequences containing PS00079 close to C-terminus also had PS00080 which overlapped with PS00079. It was reported that PS00079 may detect domains without copper-binding ability, while PS00080 could be specific to the copper-binding activity. In the motif near the C-terminus, the first two histidines (positions 6 and 8) were copper type 3 binding residues, while C, the third H, and L or M (positions 7, 12, and 17) were copper type 1 ligands [41,72,73] (lower panel in Figure 3(C)). It was shown that our functional classification was reflected in protein sequences and also in agreement with the previous reports.

There were two lignin-associated groups, the subclade A1 and clade D, which were specific to the phyla Basidiomycota and Ascomycota, respectively (Figure 2). Interestingly there was no clade of which members were solely belonging to white-rot fungi, even if they shared the unique function of lignin degradation. In addition, multiple laccases of white-rot fungi showed high sequence similarity with those of brown-rot fungi, yet no clade only comprised of rot fungi was observed. Meanwhile, it was reported that brown-rot basidiomycetes or some rot ascomycetes could only modify the side chain or the ring of lignin, while white-rot fungi could directly degrade them [74,75]. Considering that higher secretory potential of the clade D, especially for the subclade D2, than the clades B and C (Supplementary Table 3), it could be conjectured that a part of them may play roles in delignification at the interface between fungal and plant cells.

We developed an identification pipeline and database of fungal laccases and MCOs for comparative and phylogenetic analysis (http://laccase.riceblast.snu.ac.kr/). The database offers a fungal kingdom-wide archive of laccase-/MCO-encoding genes as well as those from bacteria, plants, and animal genomes for comparative analysis. Phylogenetic analysis on the representative dataset revealed functional categories, domain distribution across the taxonomy, and motif diversification of the predicted laccases and MCOs at the sequence level. Our phylogenetic analysis coupled with the database would serve as a valuable resource for comparative and evolutionary analysis of fungal laccases and MCOs.

## Supplementary Material

Supplemental Material
