# Supplementary material for: Comparative Genomics Platform and Phylogenetic Analysis of Fungal Laccases and Multi-Copper Oxidases
Source: Mycobiology. 2020 Sep 11;48(5):373–82. doi: 10.1080/12298093.2020.1816151 (PMC7594830; doi:10.1080/12298093.2020.1816151)
Supplement: Supplemental Material [file TMYB_A_1816151_SM5513.zip › Supplementary Figure 1 caption.docx]

**Supplementary Figure 1. Prediction of secretory proteins from the representative dataset.** A total of 505 laccase sequences in the representative dataset were subjected to prediction of secretory proteins by using five bioinformatics software.
