# Supplementary figures and images for: Comparative Genomics Platform and Phylogenetic Analysis of Fungal Laccases and Multi-Copper Oxidases
Source: Mycobiology. 2020 Sep 11;48(5):373–82. doi: 10.1080/12298093.2020.1816151 (PMC7594830; doi:10.1080/12298093.2020.1816151)

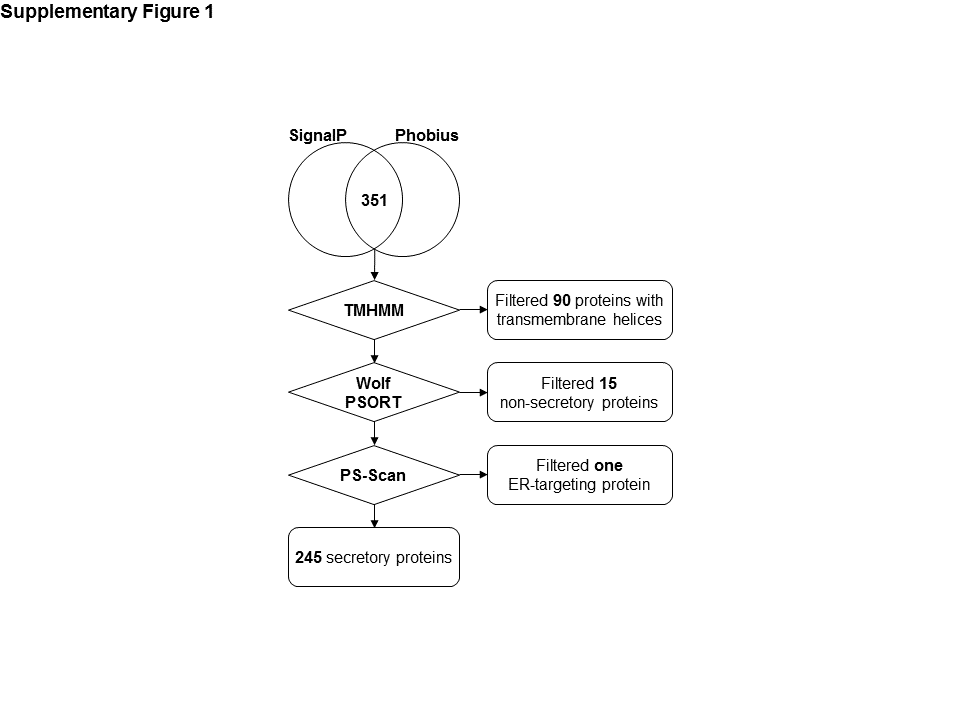

Supplement: Supplemental Material [file TMYB_A_1816151_SM5513.zip › SUPP FIG1.PNG]
